# Drug-adapted cancer cell lines as preclinical models of acquired resistance

**DOI:** 10.20517/cdr.2019.005

**Published:** 2019-09-19

**Authors:** Martin Michaelis, Mark N. Wass, Jindrich Cinatl

**Affiliations:** ^1^School of Biosciences, University of Kent, Canterbury CT2 7NJ, UK.; ^2^Institut für Medizinische Virologie, Klinikum der Goethe-Universität, Frankfurt am Main, Germany.

**Keywords:** Cancer, acquired drug resistance, cancer cell lines, drug adaptation, cancer therapy, cancer models

## Abstract

Acquired resistance formation limits the efficacy of anti-cancer therapies. Acquired and intrinsic resistance differ conceptually. Acquired resistance is the consequence of directed evolution, whereas intrinsic resistance depends on the (stochastic) presence of pre-existing resistance mechanisms. Preclinical model systems are needed to study acquired drug resistance because they enable: (1) in depth functional studies; (2) the investigation of non-standard treatments for a certain disease condition (which is necessary to identify small groups of responders); and (3) the comparison of multiple therapies in the same system. Hence, they complement data derived from clinical trials and clinical specimens, including liquid biopsies. Many groups have successfully used drug-adapted cancer cell lines to identify and elucidate clinically relevant resistance mechanisms to targeted and cytotoxic anti-cancer drugs. Hence, we argue that drug-adapted cancer cell lines represent a preclinical model system in their own right that is complementary to other preclinical model systems and clinical data.

## Introduction

Despite improvements in therapy outcomes in recent decades, except for a few exceptions (e.g., testicular cancer, Hodgkin’s lymphoma, childhood acute lymphoblastic leukaemia) cure rates remain low for advanced cancers that require systemic therapy, typically metastatic disease. In such advanced cases, the impetus typically lies on the prolongation of life and the improvement of quality of life^[1-8]^.

**Figure 1:**
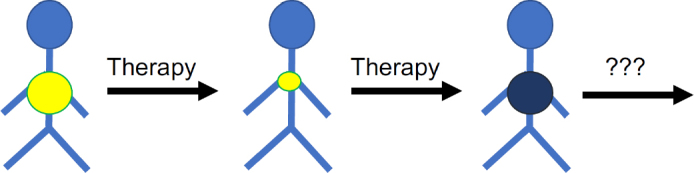
Many cancer diseases respond initially well to therapy but cancer cells become eventually resistant to therapy. An improved understanding of the mechanisms and processes underlying resistance formation is necessary to identify biomarkers that guide the use of efficient next-line therapies for tumours that have do not respond to the available standard therapies anymore

The efficacy of systemic anti-cancer therapies is limited by the occurrence of resistance. Resistance can be “intrinsic” or “upfront”, i.e., cancer cells do not respond to therapy from the outset. Many cancer diseases, however, initially respond well to therapy, but after a temporary response resistant cancer cells emerge leading to “acquired” resistance, ultimately resulting in therapy failure and patient death^[8-19]^. Hence, cancer diseases that have become resistant to the available treatment options represent an unmet clinical need. New strategies including new biomarkers that indicate effective follow-up therapies (on an individualised basis) are needed for such patients for which no established therapy options are available anymore [Figure 1].

## Intrinsic and acquired resistance mechanisms may differ

There is a conceptional difference between the mechanisms and processes underlying intrinsic and acquired drug resistance formation. Intrinsic resistance is the consequence of pre-existing, potentially stochastic changes that render cancer cells insensitive to the standard treatment. In contrast, acquired resistance is the consequence of selection and adaptation processes in response to therapy, i.e., of directed evolution induced by the therapy. In line with this, differences have been described between intrinsic and acquired resistance mechanisms^[20-23]^. Hence, acquired resistance needs to be studied in the context of the underlying (co)-evolutionary processes to establish a specific systems level understanding.

## Preclinical model systems are needed to deliver biomarkers for the effective use of “liquid biopsies” for therapy monitoring

The systematic elucidation of resistance formation depends on the combined use of preclinical model systems in combination with clinical data and specimens. Preclinical model systems enable in-depth functional and systems level studies that are difficult or impossible to perform using primary cancer cells, tissues, and/or organoids. In addition, non-standard treatments can be systematically investigated in preclinical model systems. This is not possible in a clinical setting, where patients receive standard therapies that provide the highest probability of treatment success. Hence, biomarkers that: (1) identify (small) groups of patients that are unlikely to respond to standard therapies; and (2) guide the use of more promising therapies to such patients need to be derived from preclinical models. Finally, preclinical model systems enable the direct comparison of different therapies in the same system. Such comparisons are not possible in the clinics, where every patient can only be treated once.

So-called “liquid biopsies” including circulating tumour DNA and circulating tumour cells enable the monitoring of cancer evolution and therapy response in ever greater detail^[24]^. The clinical implementation of liquid biopsies still faces many technological and methodological challenges^[24,25]^. However, the first FDA-approved assays based on liquid biopsies are available and have been shown to improve therapy outcomes^[24,26-32]^.

With the advancement of liquid biopsies for the monitoring of cancer cell evolution, a much more advanced understanding of the processes underlying therapy response and resistance formation will be required to make effective use of the wealth of omics data derived from liquid biopsies. Only an in-depth molecular understanding will enable the identification of biomarkers that indicate therapy failure early and inform the choice of effective next-line therapies. Such knowledge and the associated (putative) biomarkers will have to originate, at least in part, from research performed in preclinical model systems before they are tested in a clinical setting.

## Drug-adapted cancer cell lines reflect clinical resistance mechanisms

Cancer cell lines are among the most commonly used pre-clinical models^[33,34]^. They are relatively easy to handle and enable high throughput analysis at relatively low cost and in a timely fashion. There is increasing agreement that the use of (larger) cell line panels improves the value of results^[34,35]^. The NCI60 panel of the National Cancer Institute is the oldest and best characterised cancer cell line panel, which has contributed to the discovery of many anti-cancer drugs^[36,37]^. If typical caveats such as cell line cross-contamination and misauthentication as well as mycoplasma contamination^[38]^ are avoided, the investigation of cancer cell lines provides substantial information on cancer cell biology and drug sensitivity, as, for example, confirmed by large pharmacogenomic screens including the Genomics of Drug Sensitivity in Cancer, the Cancer Cell Line Encyclopedia, and the Cancer Therapeutics Response Portal^[39-45]^. Since most cancer cell lines have been derived from patients at diagnosis, however, they primarily reflect intrinsic resistance.

**Figure 2:**
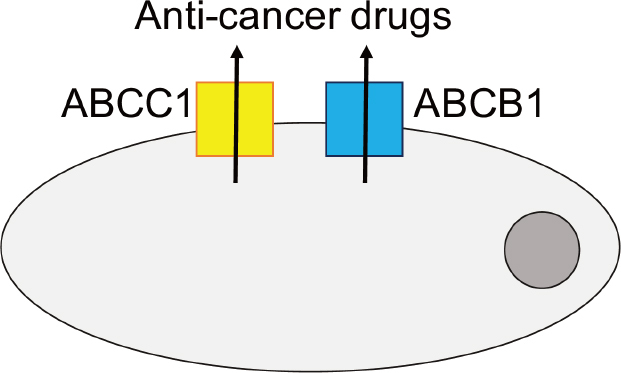
Members of the ATP-binding cassette (ABC) transporter family, including ABCB1 and ABCC1 as prominent members, belong to the most important mediators of drug resistance in cancer. Various members of the ABC transporter family function as efflux pumps that remove (often a wide range of structurally different anti-cancer drugs) from cancer cells and interfere with the achievement of effective intracellular drug concentrations

Drug-adapted cell lines better reflect the evolutionary processes leading to resistance formation. They have enabled the discovery of major drug resistance mechanisms and the identification and elucidation of clinically relevant acquired resistance mechanisms to targeted and cytotoxic anti-cancer drugs^[33]^. The ATP-binding cassette (ABC) transporters, arguably the most important mediators of drug resistance in cancer cells [Figure 2], were detected in drug-adapted cells. ABCB1 (also known as P-glycoprotein or MDR1) was discovered as the first member of the family of ABC transporters in colchicine-adapted Chinese hamster ovarian cells^[46]^. It is a promiscuous efflux pump that transports a wide range of structurally different substrates and provides resistance to a large number of anti-cancer drugs from various classes^[47,48]^. ABCC1 (also known as MRP1), another member of the ABC transporter family, is also of high importance as a cancer cell resistance mechanism^[47,48]^ and was identified in a doxorubicin-adapted subline of the lung cancer cell line H69^[49]^.

Without intending to provide a comprehensive overview, we have selected a few studies that illustrate the potential of drug-adapted cancer cell lines to reveal clinically relevant resistance mechanisms. Non-small cell lung cancer patients, who harbour cancer cells characterised by activating *EGFR* mutations, are treated with EGFR tyrosine kinase inhibitors^[50]^. In a landmark study, *MET* amplification was discovered as a resistance mechanism in a gefitinib-adapted subline of the *EGFR* exon 19 mutant non-small cell lung cancer cell line HCC827^[51]^. Further investigation of resistance formation to EGFR tyrosine kinase inhibitors using drug-adapted non-small cell lung cancer cell lines revealed that the origin of the resistance-mediating T790M *EGFR* mutation may differ in different cell line systems and patients^[52]^. Pre-existing T790M mutant subpopulations can either be selected, or *de novo* T790M mutations can be induced. The mode of resistance formation shaped the resistance phenotype of the resulting drug-resistant sublines. Induction of *de novo* T790M mutations, but not selection of pre-existing T790M mutant clones, was associated with an enhanced cellular resistance to apoptosis, which was caused by an increase in the cellular levels of anti-apoptotic bcl-2 proteins^[52]^. Furthermore, erlotinib-resistant colonies derived from non-small cell lung cancer cell lines reflected clinically observed resistance mechanisms^[53]^.

Drug-adapted cancer cell lines have also been shown to reflect clinical resistance formation to other kinase inhibitors that target specific oncogenic driver events. Inhibitors that specifically target constitutively active oncogenic V600E-mutant BRAF, have improved the therapy of melanoma patients whose tumours consist of cells that harbour V600E BRAF mutations. Unfortunately, responses are short-lived, and resistance formation is inevitable^[54]^. Key acquired resistance mechanisms to V600E-specific BRAF inhibitors including NRAS mutation, BRAF amplification, dimerization of aberrantly spliced V600E-mutant BRAF, and PDGFRB upregulation were all identified in drug-adapted cancer cell lines^[55-57]^. Moreover, clinically 5 relevant resistance mechanisms were represented in EGFR, HER2, and ALK inhibitor-adapted cancer cell lines^[58,59]^.

Drug-adapted cancer cell lines also reflect clinical resistance formation against various other “targeted” anti-cancer drugs that interfere with features that are exclusively or predominantly found in cancer cells, as demonstrated by the following examples. Prostate cancer cell lines adapted to the antiandrogen enzalutamide enabled the identification of F876L mutations in the androgen receptor as a clinically relevant resistance mechanism^[60,61]^. MDM2 inhibitors are under development as a novel class of anti-cancer drugs for the treatment of *TP53* wild-type cancer cells from different cancer entities. *TP53* encodes p53, a major tumour suppressor protein. *MDM2* is a p53 target gene that encodes for MDM2, a major endogenous inhibitor of p53. MDM2 physically interacts with p53 and mediates its ubiquitination and proteasomal degradation. MDM2 inhibitors activate p53 signalling by interference with the MDM2/p53 interaction^[62-64]^. Adaptation of *TP53* wild-type cancer cell lines has been associated with the formation of loss-of-function *TP53* mutations in many model systems^[65-70]^. In agreement, MDM2 inhibitor treatment of liposarcoma patients was associated with the emergence of *TP53* mutations^[71]^.

Drug-adapted cancer cell lines are also used to elucidate resistance mechanisms to cytotoxic anti-cancer agents. A subfraction of cells that critically depend on notch- and hedgehog signalling have been shown to be critically involved in resistance formation to doxorubicin in castration-resistant prostate cancer cells^[72]^. A number of recent studies investigated resistance formation in acute myeloid leukaemia cells using drug-adapted cell lines and identified GLI1, EZH2, and SAMHD1 as clinically relevant resistance mechanisms to cytarabine-based therapies^[73-75]^. In addition, increased glucocorticoid sensitivity was detected in cytarabine-adapted acute myeloid leukaemia cell lines and patient samples^[76]^. The use of drug-adapted cell lines has also shown that acquired resistance to cytotoxic drugs can be associated with decreased sensitivity to kinase inhibitors^[77,78]^. The clinical impact of this is difficult to determine, however, because the baseline sensitivity of tumours to different anti-cancer therapies prior to the first-line treatment is not typically known.

## Multiple resistance models are needed to reflect the heterogeneity of the processes associated with resistance formation

It is now generally accepted that cancer diseases are associated with tremendous intra-tumour heterogeneity^[79-81]^. Although therapy-induced heterogeneity has not been investigated to the same extent, there are indications that the processes underlying resistance formation are likely to be as complex^[52,82-88]^.

The advantage of cancer cell lines as models is that they are relatively easy to handle and enable high throughput analysis at relatively low cost and in a timely fashion. Although they do not reflect the original heterogeneity of the tumour they have been derived from, they are not as homogenous or clonal as previously believed^[34-37,89]^. Resistance can occur by selection of pre-existing drug-resistant subpopulations or by adaptation of originally drug-sensitive cells to anti-cancer therapies. Both mechanisms have been shown to be represented in drug-adapted cancer cell lines^[52,66-70,90-101]^.

In this context, we have adapted the *TP53* wild-type acute myeloid leukaemia (AML) cell lines MV4-11, OCI-AML-2, OCI-AML-3, and SIG-M5 to the MDM2 inhibitor nutlin-3 in multiple independent experiments^[102]^. Nutlin-3-adapted sublines of the same AML cell lines displayed a substantial heterogeneity in the response to other anti-cancer drugs. Notably, the biggest fold change (11.4) was detected in the response of two nutlin-3-adapted MV4-11 sublines to doxorubicin, although nutlin-3 treatment selected a pre-existing *TP53* mutant subpopulation in this cell line. This indicates that even the drug-induced selection of a defined pre-existing subpopulation in a cell line can result in phenotypically different sublines^[102]^. New technologies including single cell approaches will enable the elucidation of selection and adaptation processes during resistance formation in more detail^[94,103,104]^.

Since many models will be needed to cover the complexity associated with acquired resistance formation, we have established the Resistant Cancer Cell Line collection by adapting initially chemosensitive cancer cell lines to clinical concentrations of targeted and cytotoxic anti-cancer drugs to enable the systematic investigation of acquired drug resistance mechanisms. It currently contains 1300 cancer cell lines based on 125 parental cell lines from 16 cancer entities and reflects acquired resistance to 67 drugs (https://research.kent.ac.uk/ibc/the-resistant-cancer-cell-line-rccl-collection). The DEN50-R platform is another project dedicated to the generation of drug-adapted cancer cell line panels (http://www.den50-r.org).

## Conclusion

This perspective is focused on the use of drug-adapted cancer cell lines as models of acquired drug resistance in cancer. Drug-adapted cancer cell lines are, like every model system, associated with specific advantages and limitations. Models including primary cancer cell cultures, three-dimensional cell (co-)culture systems, tumour-derived organoids, and animal models better reflect certain aspects of tumour growth such as intra-tumour heterogeneity, three-dimensional architecture, cancer cell interaction with the cancer microenvironment, and/ or metastatic behaviour^[105-114]^. Such models can be used to study processes that cannot be studied in cell lines. In this context, acquired resistance models have been established based on cell line- and patient-derived xenografts, organoids, and transgenic tumour models^[115-125]^. However, cell lines enable the establishment of a substantially larger number of models within a given timeframe and at a given cost, which is critical for studying the drug-induced heterogeneity. Notably, data so far suggest that the drug adaptation of cancer cell lines reveals similar resistance mechanisms as cell line-derived xenografts and transgenic mouse models^[116,118,123,125]^.

**Figure 3:**
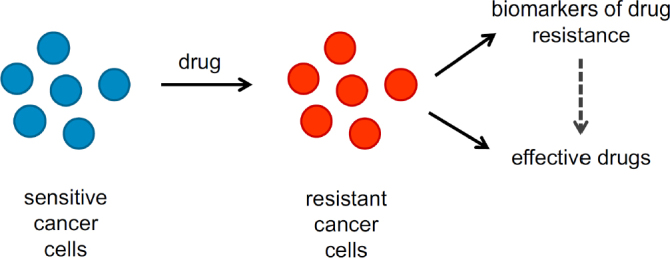
Drug-adapted cancer cell lines enable the identification of candidate biomarkers that enable the early detection of resistance formation and, in combination with drug screens and functional genomics approaches, the selection of effective next-line therapies

In conclusion, drug-adapted cancer cell lines reflect clinically relevant acquired drug resistance mechanisms and represent a preclinical model system in their own right, which is complementary to other preclinical models and clinical specimens. Drug-adapted cancer cell lines enable systems level studies and the direct comparison of different therapies in the same system that cannot be performed in the clinics. Hence, drug-adapted cancer cell lines offer potential for the identification of biomarkers that indicate resistance formation and, ideally, effective next-line therapies [Figure 3]. Many drug-adapted cancer cell lines will be needed to cover the complexity of the mechanisms underlying resistance formation.

## Declarations

### Copyright

© The Author(s) 2019.
